# Retraction Note: Mir-655 up-regulation suppresses cell invasion by targeting pituitary tumor-transforming gene-1 in esophageal squamous cell carcinoma

**DOI:** 10.1186/s12967-020-02265-4

**Published:** 2020-02-17

**Authors:** Yuanyuan Wang, Wenqiao Zang, Yuwen Du, Yunyun Ma, Min Li, Ping Li, Xudong Chen, Tao Wang, Ziming Dong, Guoqiang Zhao

**Affiliations:** 1 College of Basic Medical Sciences, Zhengzhou University, No.100 Kexue Road, Zhengzhou, 450001 Henan China; 2 Department of Respiratory Medicine, The First Affiliated Hospital of Zhengzhou University, Zhengzhou, 450052 Henan China; 3 Department of Histology and Embryology, Luohe Medical College, Luohe, 462002 Henan China; 4 Department of Hemato-tumor, The First Affiliated Hospital of Henan University of TCM, Zhengzhou, 450000 Henan China

## Retraction Note: J Transl Med (2013) 11:301 10.1186/1479-5876-11-301

The Editor-in-Chief has retracted this article [1] because Figure 3a overlaps with Figure 2 in [2]. An investigation by Zhengzhou University has confirmed this. The data reported in this article are therefore unreliable. There is also considerable text overlap with a previously published article [3]. Guoqiang Zhao does not agree with this retraction. The other authors have not responded to correspondence from the editor about this retraction.
